# Veterinary Inspection at Horse Shows

**Published:** 1892-06

**Authors:** 


### EDITORIAL.

### VETERINARY INSPECTION AT HORSE SHOWS.

It is the custom at horse shows to have a board of veterinarians in attendance. The object of such a board is not only for the care of the animals, but more especially to pass upon the soundness or unsoundness of the animals exhibited. That such a board should be composed of members in good standing in their profession goes without saying, for upon their opinion is often based the decision of the judges after the competition has been narrowed down to a few animals.

That method in these show-ring examinations is as necessary as the opportunities for examination are lacking, is evident. How to arrange a method is the question.

Just how far should the duties of veterinarian extend? Should he simply pass upon the actual soundness or unsoundness of an animal at the time, or should he call the attention of the judges to defects in conformation? Should an animal be accompanied by the certificate of the attending veterinarian as to soundness or unsoundness, when he comes to the show, as an aid to the Veterinary Inspector, or should such certificates be ignored? Should utility animals and breeding animals stand upon the same footing and be submitted to the same examination, or should the examination for breeding animals be much more rigid? Should all classes of animals be examined in a paddock, especially arranged for the purpose, before allowing them to go into the ring, or should this apply only to breeding classes? Such were the questions which arose in the minds of the veterinarians, judges and executive committee at the show just finished in New York, and in answer to them the writer offers the following as his opinion:

1. It would be well if a veterinary certificate as to soundness or unsoundness were to accompany each animal.

2. All animals should be examined by the veterinarians at the show, whether accompanied by veterinary certificate or not.

3. All animals intended for prize competition should be on the ground and at rest for at least three hours previous to examination for soundness, so that no advantage may be gained by submitting animals for examination while warm.

4. All animals should be examined in a paddock, built for the purpose, before entering the ring.

5. The paddock should be closed in and should have two runs, one of stone and the other of dirt.

6. The horses should be attended while in the paddock by grooms in the employ of the association, and the record should be endorsed with the number of the horse, by the clerk, when the examination is finished.

7. The veterinarian should make note not only of the unsoundness, but should call attention to any defects of conformation which, in his opinion, would make the animal susceptible to any especial disease.

8. The veterinarians should be *ex-officio*, members of each board of judges, and should be in the ring to detect lameness or any defect in breathing, which it may not have been possible to detect in the paddock, and to advise the judges as to how much importance should be attached to any lameness which developed in the ring.

9. Three sets of rules shold govern the veterinarian in his examinations.

(*a.*) Breeding animals should be absolutely free from hereditary unsoundness or from such defective conformation as would, from slight exciting causes, develop an unsoundness.

(*b.*) Saddle and harness horses should be practically sound to be eligible for a prize, and only when the competition is narrowed down to a few animals should the question of absolute soundness be considered

(*c.*) Hunters intended only as such should go sound.

To the honesty and ability of the veterinarian is due, in a great measure, the success of a show. His opinion should, therefore, carry weight with the judges, and he should be given every possible facility for conducting his examinations. A. W. C.

